# 异基因造血干细胞移植治疗难治/复发弥漫大B细胞淋巴瘤的作用及其进展

**DOI:** 10.3760/cma.j.cn121090-20231017-00213

**Published:** 2024-03

**Authors:** 乐清 曹, 晓冬 莫

**Affiliations:** 北京大学人民医院，北京大学血液病研究所，国家血液系统疾病临床医学研究中心，造血干细胞移植治疗血液病北京市重点实验室，北京 100044 Peking University People's Hospital, Peking University Institute of Hematology, National Clinical Research Center for Hematology Diseases, Beijing Key Laboratory of Hematoietic Stem Cell Transplanlalion, Beijing 100044, China

## Abstract

弥漫大B细胞淋巴瘤（DLBCL）是最常见的非霍奇金淋巴瘤类型。目前的治疗方案能显著改善患者的预后，但30％～40％的DLBCL患者出现难治（耐药）或复发。对于难治/复发的DLBCL患者，临床上治疗仍困难，其预后不佳。异基因造血干细胞移植（allo-HSCT）仍是难治/复发DLBCL患者最重要的根治手段之一。本文就allo-HSCT治疗难治/复发DLBCL作用及其进展进行综述。

## Abstract (en)

Diffuse large B-cell lymphoma（DLBCL）is the most common type of non Hodgkin's lymphoma. The current treatment plan can significantly improve the prognosis of patients, but about 30％–40％ of DLBCL patients still experience drug resistance and relapse after treatment. For patients with refractory/relapse DLBCL, clinical treatment remains difficult and their prognosis is poor. Allogeneic hematopoietic stem cell transplantation（allo-HSCT）remains one of the most important curative methods for refractory/relapse DLBCL patients. This article will review the role and progress of allo-HSCT in the treatment of refractory/relapse DLBCL.

弥漫大B细胞淋巴瘤（diffuse large B-cell lymphoma, DLBCL）是最常见的非霍奇金淋巴瘤类型。Sun等[1]对4 638例淋巴瘤病理类型的统计发现，DLBCL占所有非霍奇金淋巴瘤的39.6％。Yang等[2]对6 382例非霍奇金淋巴瘤病理类型的统计显示DLBCL分别占结内病变、结外病变的39.8％、42.5％。在DLBCL的患者中，采用利妥昔单抗联合传统化疗的治疗方案可显著改善患者的预后，约70％的患者可获得完全缓解（CR）/未确定的完全缓解[3]，这些患者10年无病生存率可达60％以上[4]；近年来以维泊妥珠单抗为基础的治疗方案更是将患者的2年无进展生存（DFS）率提高到70％[5]–[6]。然而仍有30％～40％的DLBCL患者会出现难治（耐药）或复发，对于难治/复发的DLBCL患者，临床上治疗仍困难，其预后不佳[7]–[8]。目前关于难治DLBCL尚无统一的定义，Coiffier等[9]定义为对一线治疗无反应，或在CR后12个月内复发；Cheson等[10]定义为对二线/三线治疗无反应，或二线/三线治疗获得CR/部分缓解（PR）后6个月内发生疾病进展；Hitz等[11]定义原发难治性DLBCL为对一线治疗无反应，或达到CR/PR后3个月内复发；SCHOLAR-1研究[12]定义为化疗后疾病进展或疾病稳定为最佳反应，或自体造血干细胞移植（auto-HSCT）后12个月内复发。我国一项纳入2778例DLBCL的多中心回顾性研究认为SCHOLAR-1研究的定义最适用于确定难治性DLBCL的患者[8]。Crump等[12]统计了636例难治/复发DLBCL患者的转归发现，接受挽救治疗的总反应率为26％，其中CR率仅为7％，中位生存时间仅6.3个月。迄今为止，虽然涌现了不少新的治疗模式，但异基因造血干细胞移植（allo-HSCT）仍是难治/复发DLBCL患者最重要的根治手段之一。本文就allo-HSCT治疗难治/复发DLBCL作用及其进展进行综述。

一、allo-HSCT可作为auto-HSCT失败后的重要挽救治疗

auto-HSCT是年轻、化疗敏感的难治/复发DLBCL患者重要挽救治疗手段。欧洲骨髓移植登记组共纳入6 717例接受auto-HSCT的难治/复发DLBCL患者，其中80％为化疗敏感，20％为化疗耐药，分析其资料显示患者移植后100 d和4年非复发死亡率（NRM）分别为3％、7％，4年复发率、无进展生存（PFS）率、总生存（OS）率分别为50％、43％、54％，多因素分析显示高龄、诊断1年内移植以及化疗耐药与不良预后相关[13]。

对于auto-HSCT后难治/复发的DLBCL患者，allo-HSCT是有效的挽救治疗手段。欧洲骨髓移植登记组的资料显示，101例auto-HSCT后复发的DLBCL患者选择同胞相合或非血缘供者allo-HSCT，移植后3年疾病进展率为30.1％，NRM为28.2％，OS率、PFS率分别为53.8％、41.7％[14]。

早期也有研究比较了auto-HSCT和allo-HSCT在难治/复发DLBCL患者中的作用。国际骨髓移植登记组报道了916例接受造血干细胞移植的DLBCL患者的资料（auto-HSCT组836例，同胞相合allo-HSCT组79例），结果显示allo-HSCT组患者移植后1年、3年和5年疾病复发/进展率分别为30％、33％和33％，低于auto-HSCT组的33％、37％和40％；但allo-HSCT组移植后1、3、5年的NRM分别为41％、43％、45％，均高于auto-HSCT组（分别为12％、16％、18％），因此allo-HSCT组无论是PFS率还是OS率均低于auto-HSCT组[15]。来自欧洲骨髓移植登记组的资料共纳入2002年至2009年共4 210例诊断为DLBCL的患者，比较了auto-HSCT（3 980例）、清髓allo-HSCT（132例）以及减低强度预处理allo-HSCT（98例）治疗难治/复发DLBCL患者的临床结局，相应的患者4年NRM分别为7％、25％和19％，4年复发率分别为50％、47％和53％，4年PFS分别为43％、28％和28％，4年OS率分别为54％、30％和38％[13]。从上述大宗病例研究中可以看到，相较于auto-HSCT，allo-HSCT组患者的复发率与其接近，而NRM明显增高，从而导致患者的PFS和OS明显减低。因此，allo-HSCT无法取代auto-HSCT的地位，因而被推荐作为auto-HSCT后复发/进展的DLBCL患者的挽救治疗。

二、allo-HSCT作为嵌合抗原受体T细胞（CAR-T）治疗失败的挽救治疗

CAR-T治疗的问世给难治/复发DLBCL患者的治疗带来了革命性进步，是难治/复发DLBCL（尤其是12个月内的早期复发以及原发耐药DLBCL）患者最重要的挽救治疗手段之一。在JULIET研究[16]中，CAR-T治疗最佳总体治疗反应率为52％，完全反应率达到40％，1年OS率为49％。在TRANSCEND NHL 001研究[17]中，CAR-T治疗后总反应率达73％，完全反应率达53％，1年OS率、PFS率分别为58％、44％。在近期公布的国际多中心随机对照研究（ZUMA-7研究）[18]中，CAR-T治疗组总反应率、完全反应率分别为83％、50％，无事件生存时间为8.3个月，优于以auto-HSCT为基础的对照组。但在BELINDA研究中，CAR-T组和以auto-HSCT为基础的对照组相比无论是治疗反应率（75％对68％）还是无事件生存时间（中位均为3个月）等均无显著差异[19]。由于ZUMA-7研究不纳入快速进展或合并巨大肿块的患者，有可能人为排除了部分预后不佳的DLBCL患者，而BELINDA则允许纳入上述患者，或许更能反映CAR-T在真实世界中对于难治/复发DLBCL患者的疗效[20]。这也提示很多难治/复发DLBCL患者未能在CAR-T后获得持久的疾病控制。

真实世界研究也表明CAR-T治疗后的远期疗效似乎并不那么令人满意。在德国的真实世界研究中（GLA/DRST研究），356例接受CAR-T治疗的难治/复发DLBCL患者，1年复发率达到67％，PFS率仅为30％[21]。在西班牙GELTAMO/GETH-TC工作组的真实世界研究中，接受CAR-T治疗的难治/复发DLBCL患者中位PFS时间仅为4.6个月，1年PFS率为28％且PFS曲线未出现平台[22]。在美国的真实世界研究中，275例接受CAR-T治疗的难治/复发DLBCL患者，回输后中位无进展生存时间也仅有8.31个月，回输后12个月PFS率仅47％[23]。

CAR-T治疗后进展，尤其是治疗后30 d内进展的患者预后极差[24]，对于这些患者来说，allo-HSCT仍是最重要的挽救治疗手段。Shadman等[25]分析了CAR-T治疗失败的非霍奇金淋巴瘤患者接受挽救性allo-HSCT的安全性，移植后Ⅱ～Ⅳ度和Ⅲ/Ⅳ度急性移植物抗宿主病（GVHD）的发生率分别为77％和31％，仅1例发生慢性GVHD，1年NRM和OS率分别33％和59％。Zurko等[26]近期公布了allo-HSCT作为CAR-T治疗失败的DLBCL患者挽救治疗的临床数据，88例患者中9例直接进行allo-HSCT，其余79例患者在其他挽救治疗后接受allo-HSCT，移植后1年复发/进展率、NRM、PFS率、OS率、无GVHD/无复发生存率分别为33％、22％、45％、59％和39％。这表明，对于CAR-T治疗失败的患者，接受allo-HSCT仍有机会获得持久的疾病控制。

三、auto-HSCT失败的DLBCL患者，allo-HSCT和CAR-T治疗疗效比较？

对于auto-HSCT失败的DLBCL患者，allo-HSCT和CAR-T治疗都是有效的挽救治疗手段。Hamadani等[27]的研究纳入2012-2019年期间接受auto-HSCT后复发（≥18岁）的DLBCL患者，其后续分别选择CAR-T（181例）或allo-HSCT（403例）治疗，CAR-T组患者治疗时间集中在2018至2019年，且该组患者更年轻、治疗前未缓解的比例更高，但随访时间较短；CAR-T组治疗后1年的复发率、NRM、OS率、PFS率分别为39.5％、4.8％、73.4％、55.7％，allo-HSCT组治疗后1年的复发率、NRM、OS率、PFS率分别为26.2％、20.0％、65.6％、53.8％；allo-HSCT组复发率较低，但NRM较高，最终两组患者的生存情况类似。与此同时，Dreger等[28]基于意向性治疗的比较也有类似结果，接受allo-HSCT、CAR-T治疗的难治/复发DLBCL患者其治疗后总反应率分别为71％、79％，1年复发率分别为44％、59％，NRM分别为21％、3％，PFS率分别为35％、36％，OS率分别为54％、68％。目前尚无随机对照研究比较这两种治疗方法在auto-HSCT失败DLBCL患者中的临床结局。基于现有临床资料，allo-HSCT和CAR-T治疗均可作为这些患者的挽救治疗手段。

四、CAR-T治疗后桥接allo-HSCT治疗难治/复发DLBCL的可行性

对于接受allo-HSCT的DLBCL患者，移植前获得CR的患者具有较好的移植后生存[26]。但对于CAR-T治疗失败的患者来说，大部分患者可能无法再次获得治疗反应。DESCAR-T研究的结果显示，CAR-T治疗后复发/疾病进展的患者接受挽救治疗总反应率仅为14％，挽救治疗措施包括双抗类药物、放疗、免疫化学治疗、靶向药物、来那度胺等，其对应的治疗反应率分别为3％、7％、8％、5％、1％[24]。即使采用维泊妥珠单抗治疗，CAR-T治疗后复发/耐药的DLBCL患者的总反应率也仅有44％（其中CR率仅为14％）[29]。目前已有研究探索了DLBCL患者接受CAR-T治疗后疾病进展相关的可能的危险因素[21],[23]。Vercellino等[30]针对DLBCL患者接受CAR-T治疗后早期进展危险因素的分析中显示，结外病灶≥2个以及总肿瘤代谢容积>80 ml是早期复发和死亡的最重要危险因素；同时具有上述两个危险因素的患者CAR-T治疗后生存情况最差。因此，对于有上述危险因素的DLBCL患者，通过CAR-T治疗获得治疗反应后及时桥接allo-HSCT或许有助患者获得更持久的疾病控制。

五、总结

在新型药物和CAR-T治疗已经蓬勃发展的现在，allo-HSCT仍是难治/复发DLBCL患者最重要的根治手段之一。新型药物目前主要包括以下几大类：双特异性抗体、抗体偶联药物、免疫检查点抑制剂和小分子药物等，是改善难治/复发DLBCL患者生存的重要进步。目前很多新型药物的疗效已在临床研究中获得验证[31]–[37]，但关于新型药物治疗获得反应后的后续治疗选择及是否桥接auto-HSCT或allo-HSCT目前尚无统一推荐。一项研究纳入了原发难治且CAR-T治疗后复发的DLBCL和原发性纵隔B细胞淋巴瘤的患者各1例，分别予维博妥珠单抗联合利妥昔单抗和苯达莫司汀（Pola-BR）治疗3个疗程获得完全代谢缓解后桥接了allo-HSCT，2例患者分别获得移植后362 d和195 d无病生存，因此认为Pola-BR可以作为CAR-T治疗后复发患者接受allo-HSCT前的有效过渡治疗[38]。对于难治/复发DLBCL患者，尤其是CAR-T治疗后仍未获得治疗反应或复发的患者，采用新型药物治疗获得治疗反应后桥接allo-HSCT以使患者获得更长生存是未来值得研究的方向。

在难治/复发DLBCL患者的治疗中，allo-HSCT和CAR-T治疗到底孰优孰劣尚难定论。CAR-T治疗虽然相对安全，早期治疗反应率高，但远期疾病控制情况尚待观察；而且CAR-T制备需要时间，商业化的CAR-T产品价格较昂贵且医保没有覆盖[39]等也是难以回避的问题。在既往研究中，allo-HSCT降低复发率所带来的生存获益往往被较高的NRM所抵消。近年来，随着移植技术的发展，针对重度GVHD、感染等移植后常见并发症的预测和治疗方法获得长足进步[40]–[45]，移植后NRM已经显著下降[46]–[47]，因此allo-HSCT仍将在难治/复发DLBCL患者的治疗中发挥重要的作用。未来设计良好的随机对照研究将有助进一步比较allo-HSCT和CAR-T在难治/复发DLBCL患者中的疗效和安全性；而对于预期CAR-T治疗后远期疗效不佳的患者，尤其对于疾病快速进展或合并巨块型病灶的DLBCL患者，allo-HSCT或许有助进一步改善患者的长期无病生存。
